# A Rare Case of Submandibular Gland Squamous Cell Carcinoma: Diagnostic Challenges and Surgical Management

**DOI:** 10.7759/cureus.52287

**Published:** 2024-01-15

**Authors:** Nikhil Thatipalli, Anup Zade, Darshana Tote

**Affiliations:** 1 Surgery, Jawaharlal Nehru Medical College, Datta Meghe Institute of Higher Education and Research, Wardha, IND; 2 Surgery, Mahatma Gandhi Institute of Medical Sciences, Wardha, IND

**Keywords:** surgical excision, multidisciplinary approach, fine needle aspiration cytology (fnac), submandibular gland, salivary glands, squamous cell carcinoma

## Abstract

Squamous cell carcinoma (SCC) in salivary glands is rare, often posing diagnostic challenges. This case report presents a 75-year-old male with progressively increasing swelling and pain in the right submandibular gland, eventually diagnosed as moderately differentiated SCC. The diagnostic journey involved fine needle aspiration cytology, imaging studies, and histopathological examination. The patient underwent surgical intervention, including submandibular gland excision and selective neck dissection, which successfully achieved local control. Subsequent postoperative follow-up indicated a favorable outcome, with no significant complaints. This report contributes insights into the multidisciplinary diagnostic approach and underscores the importance of imaging modalities in managing salivary gland SCC. This rare case emphasizes the need for ongoing research to refine management strategies for salivary gland SCC. By presenting a comprehensive diagnostic and therapeutic approach, this report contributes to the limited literature on this malignancy, emphasizing its rarity and the necessity for continued exploration of long-term outcomes. In conclusion, our case provides valuable insights into the medical knowledge surrounding SCC in salivary glands, warranting attention and further investigation.

## Introduction

Salivary gland tumors are relatively uncommon neoplasms. The average annual incidence rate is about three cases per 100,000 people in the Western world, with an average age of diagnosis at 55 years. Most salivary gland cancers are found in the parotid glands, followed by the submandibular, sublingual, and minor salivary glands. The five-year relative survival rate for salivary gland cancer in the United States is 76% [1]. While the majority of salivary gland tumors are benign, malignant tumors constitute a significant clinical concern due to their potential for aggressive behavior and the challenges associated with their management [2].

Squamous cell carcinoma (SCC) in the salivary glands is rare, representing a small fraction of salivary gland malignancies [3]. Most salivary gland tumors are of epithelial origin, with adenocarcinomas and mucoepidermoid carcinomas being the more prevalent histological types [4]. SCC is more commonly associated with the mucosa of the upper aerodigestive tract, and its occurrence within the salivary glands is distinctly infrequent [5]. Diagnostic evaluation of salivary gland lesions poses a unique challenge, necessitating a combination of clinical, radiological, and histopathological approaches. Fine needle aspiration cytology (FNAC) guided by imaging modalities such as ultrasound is a widely employed initial diagnostic tool [6]. However, due to the heterogeneity of salivary gland tumors, further investigations, including imaging studies like computed tomography (CT) and magnetic resonance imaging (MRI), are often required for a comprehensive assessment of tumor characteristics and extent [7].

The management of salivary gland SCC involves a multidisciplinary approach, with surgery as the primary modality. The choice of surgical intervention depends on factors such as tumor location, size, and histopathological features [8]. Adjuvant therapies, including radiotherapy and chemoradiotherapy, may be considered based on the risk of recurrence and the presence of adverse prognostic factors [9].

## Case presentation

**Figure 1:**
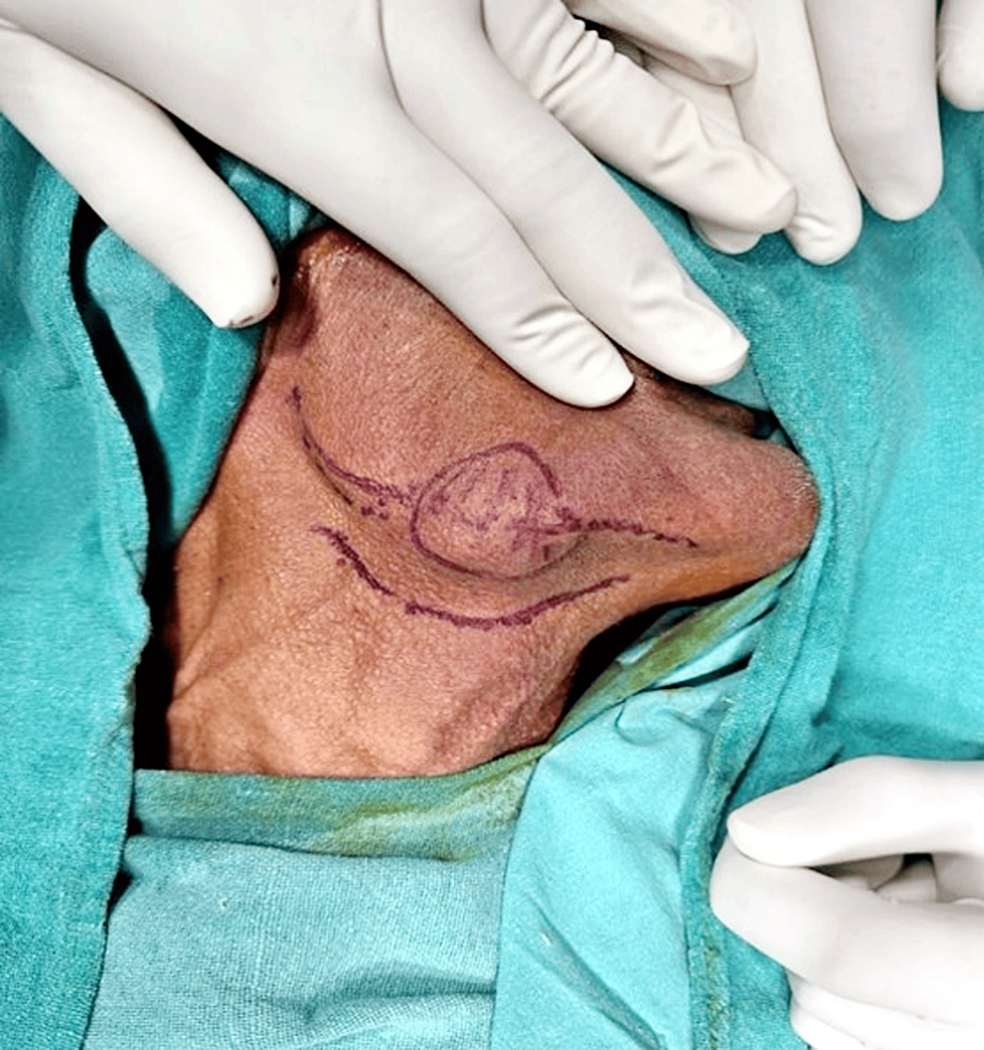
Swelling measuring 4 x 2 cm was noted in the right submandibular region.

A 75-year-old male patient presented to the department with a chief complaint of progressively increasing swelling in the right submandibular gland over the past four months, accompanied by pain. There was no history of prior neck radiation. Clinical examination of the oral cavity and neck revealed no abnormalities. A tender, firm, and fixed swelling measuring 4 x 2 cm was noted in the right submandibular region (Figure 1). No palpable lymph nodes were detected on either side of the head and neck. General examination yielded normal results, with no evidence of systemic diseases. A preliminary diagnosis of a salivary gland tumor was considered.

**Figure 2:**
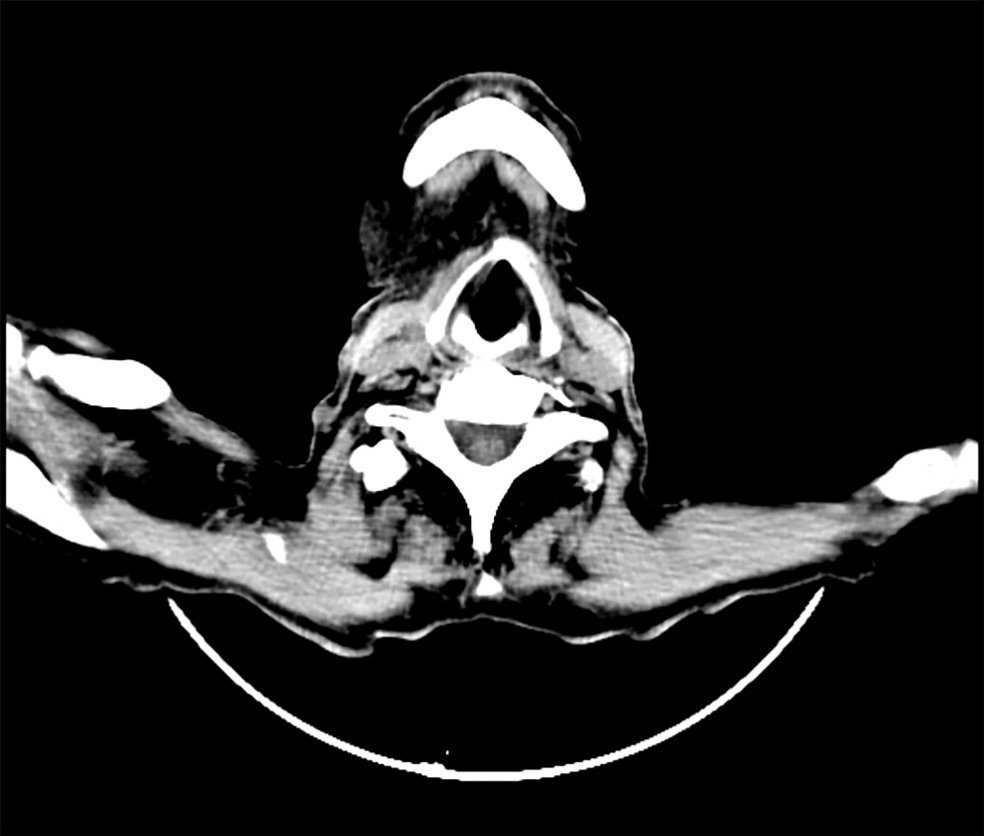
Heterogeneously enhancing irregular soft tissue density lesion in the right submandibular space.

An ultrasound-guided FNAC was performed, revealing findings suggesting well-differentiated SCC with central cystic necrosis. To rule out the primary source, a triple endoscopy and high-resolution computed tomography (HRCT) were conducted, and the results were within normal limits. Contrast-enhanced computed tomography (CECT) of the head and neck indicated a heterogeneously enhancing irregular soft tissue density lesion in the right submandibular space, measuring 4.8 x 2.4 x 2.4 cm (Figure 2), with necrotic areas. The lesion encased the right facial artery, with no evidence of adjacent bony erosion or significant cervical lymphadenopathy.

**Figure 3:**
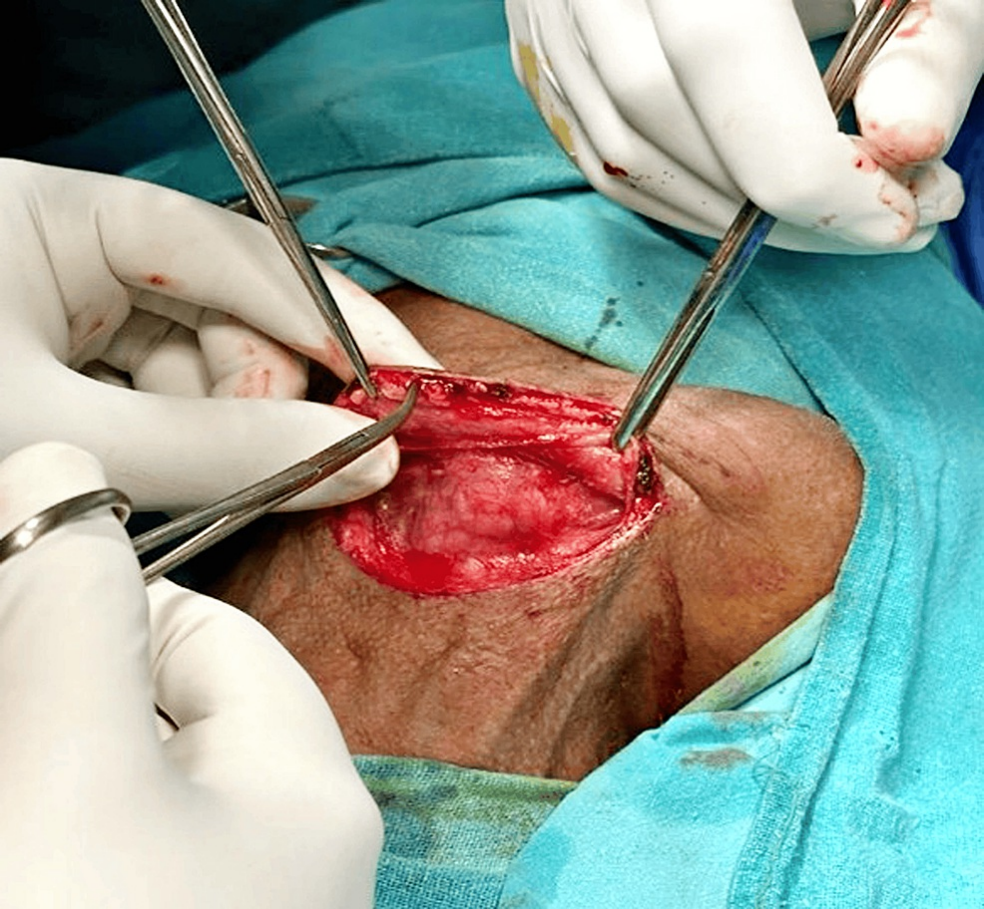
Submandibular gland excision and selective neck dissection of levels II, III, and IV were performed.

**Figure 4:**
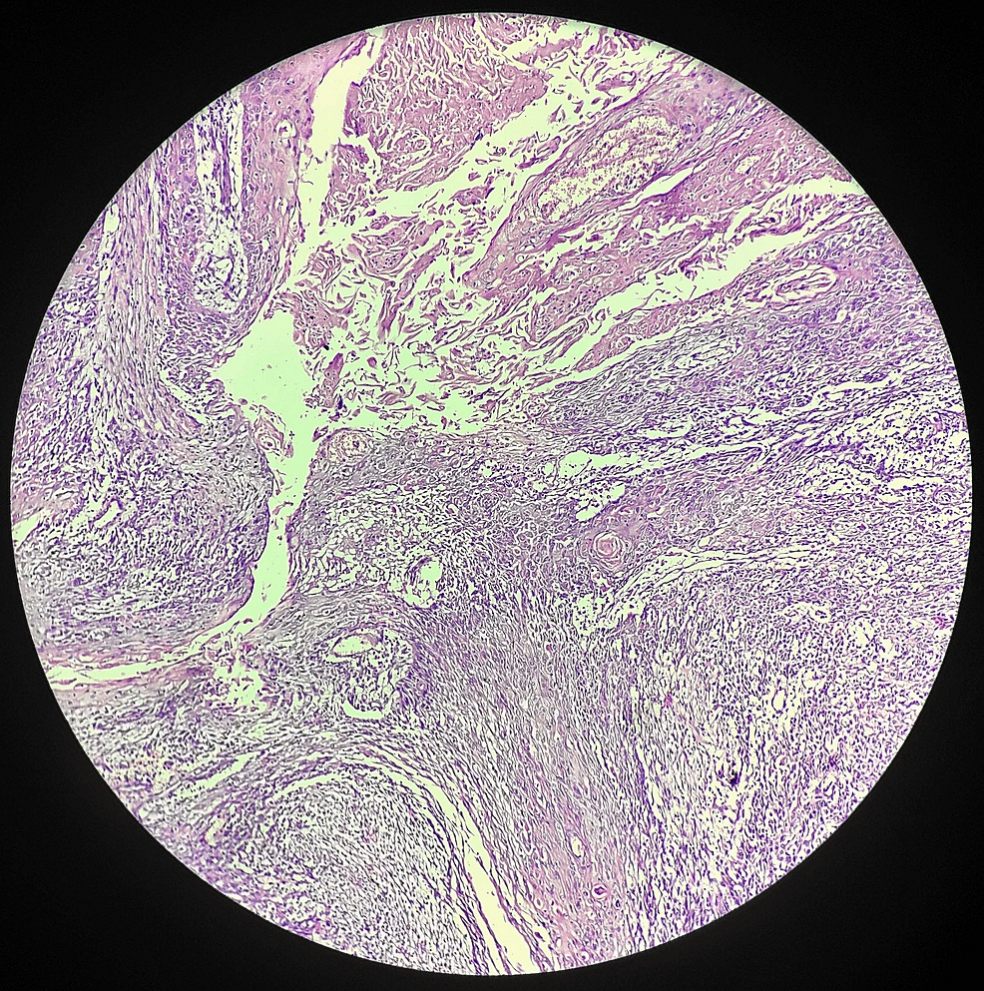
Moderately differentiated squamous cell carcinoma.

Subsequently, submandibular gland excision and selective neck dissection of levels II, III, and IV were performed (Figure 3). The excised salivary gland and lymph nodes were sent for histopathological examination. The salivary gland specimen grossly revealed a globular, greyish-brown tissue piece measuring 4 x 3 x 1.8 cm. Microscopic examination indicated moderately differentiated SCC in the submandibular gland (Figure 4). Lymph nodes showed reactive lymphoid tissue nests surrounded by fibro-collagenous tissue, with no infiltration by malignant cells on histopathology.

Following the surgical intervention involving submandibular gland excision and selective neck dissection, the patient exhibited a remarkably favorable postoperative course. Histopathological examination of the excised tissue confirmed the presence of moderately differentiated SCC in the submandibular gland. Crucially, the examination of regional lymph nodes revealed no infiltration by malignant cells, indicating a localized disease process. This absence of metastasis to the lymph nodes contributed significantly to the overall positive prognosis for the patient. Postoperative follow-up assessments showed no significant complaints, and the patient reported a notable improvement in symptoms, including reduced swelling and pain in the right submandibular region. The success of the multidisciplinary approach, encompassing surgical intervention and subsequent vigilance in postoperative care, was evident in the patient's well-being during the recovery period. The absence of complications and the encouraging clinical progress underscored the efficacy of the management strategy implemented in achieving local control and a favorable outcome for the 75-year-old male patient with SCC in the submandibular gland.

## Discussion

SCC in the salivary glands is an uncommon malignancy, accounting for a small percentage of all salivary gland tumors [10]. Most salivary gland tumors are of epithelial origin, with the majority classified as adenocarcinomas or mucoepidermoid carcinomas [4]. SCC typically arises in the mucosa of the upper aerodigestive tract, and its occurrence in the salivary glands is rare [11]. The initial presentation of swelling and pain in the submandibular gland prompted a thorough diagnostic workup. FNAC guided by ultrasound, a valuable tool in evaluating salivary gland lesions, provided initial insights into the nature of the tumor [6]. The subsequent triple endoscopy and HRCT aimed at identifying the primary source of the carcinoma yielded normal results, emphasizing the need for a comprehensive diagnostic approach.

CECT played a crucial role in delineating the characteristics of the lesion in the right submandibular space. The imaging findings indicated a heterogeneously enhancing irregular soft tissue density lesion with necrotic areas, emphasizing the need for surgical intervention [12]. Submandibular gland excision and selective neck dissection of levels II, III, and IV were performed to achieve local control and assess regional lymph node involvement. Histopathological examination of the excised tissue confirmed the presence of moderately differentiated SCC in the submandibular gland. The absence of malignant cell infiltration in the examined lymph nodes suggested a localized disease process, contributing to the overall favorable prognosis.

The management of salivary gland SCC involves a multidisciplinary approach, with surgery remaining the cornerstone. Adjuvant therapies, such as radiotherapy or chemoradiotherapy, may be considered based on the extent of the disease and histological characteristics [12]. In this case, the patient reported for regular follow-up with no significant complaints, underscoring the importance of vigilant surveillance in post-treatment care.

## Conclusions

In conclusion, the diagnostic journey, incorporating FNAC, imaging studies, and histopathological examination, facilitated a precise diagnosis, guiding subsequent surgical intervention. The comprehensive approach achieved local control, including submandibular gland excision and selective neck dissection. Histopathological confirmation of moderately differentiated SCC in the submandibular gland and the absence of malignant cell infiltration in examined lymph nodes point toward a favorable postoperative course. The lack of significant complaints during regular follow-up underscores the success of the management strategy. This case contributes valuable insights to the limited literature on salivary gland SCC, emphasizing the significance of a multidisciplinary approach and the role of imaging modalities in characterizing lesions. However, due to the rarity of this malignancy, ongoing research is essential for refining treatment approaches and understanding long-term outcomes, making this case a valuable addition to the medical knowledge on SCC in salivary glands.
